# Stimbiotic Supplementation Alleviates Poor Performance and Gut Integrity in Weaned Piglets Induced by Challenge with *E. coli*

**DOI:** 10.3390/ani12141799

**Published:** 2022-07-13

**Authors:** DongCheol Song, JiHwan Lee, WooGi Kwak, MinHo Song, HanJin Oh, YongJu Kim, JaeWoo An, SeYeon Chang, YoungBin Go, HyunAh Cho, HyeunBum Kim, JinHo Cho

**Affiliations:** 1 Department of Animal Sciences, Chungbuk National University, Cheongju 286-44, Korea; paul741@daum.net (D.S.); junenet123@naver.com (J.L.); kwakwooki@naver.com (W.K.); dhgkswls17@naver.com (H.O.); xormakzm@naver.com (Y.K.); blueswing547@naver.com (J.A.); angella2425@naver.com (S.C.); rhdudqls3@gmail.com (Y.G.); hannah0928@naver.com (H.C.); 2 Division of Animal and Dairy Science, Chungnam National University, Daejeon 341-34, Korea; mhsong@cnu.ac.kr; 3 Department of Animal Resource and Science, Dankook University, Cheonan 311-16, Korea

**Keywords:** stimbiotic, immune response, gut health, *E. coli*

## Abstract

**Simple Summary:**

Post-weaning diarrhea (PWD) caused the destruction of tight junction and epithelial cells, resulting in the increased gut permeability of pathogenic E.coli, and the reduction of the growth performance. Stimbiotic (STB), while generating specific Xylo-oligosaccharides and a positive effect on gut health, has been introduced as an antibiotics alternative. This study evaluated the effect in weaned piglets of experimentally induced PWD. Our results showed that the pigs were challenged by *Shiga toxigenic* *Escherichia coli* (STEC). STB significantly increased the growth performance, immunity and intestinal health compared with the non-supplemented group. Therefore, STB can be used as an effective additive for weaned piglets.

**Abstract:**

The aim of this study was to investigate the effects of stimbiotic (STB), a xylanase and xylo-oligosaccharide complex. A total of 36 male weaned pigs with initial body weights of 8.49 ± 0.10 kg were used in a 3-week experiment. The experiment was conducted in a 2 × 3 factorial arrangement (six replicates/treatment) of treatments consisting of two levels of challenge (challenge and non-challenge) and three levels of STB (0, 0.5, and 1 g/kg diet). Supplementations STB 0.5 g/kg (STB5) and STB 1 g/kg (STB10) improved the G:F (*p* = 0.04) in piglets challenged with STEC. STB supplementation, which also decreased (*p* < 0.05) the white blood cells, neutrophils, lymphocytes, and expression levels of tumor necrosis factor-alpha and interleukin-6. Supplementations STB5 and STB10 improved (*p* < 0.01) the lymphocytes and neutrophils in piglets challenged with STEC on 14 dpi. Additionally, supplementations STB5 and STB10 improved (*p* < 0.01) the tumor necrosis factor-alpha in piglets challenged with STEC on 3 dpi. Supplementations STB5 and STB10 also improved the villus height-to-crypt depth ratio (*p* < 0.01) in piglets challenged with STEC. Supplementation with STB reduced (*p* < 0.05) the expression levels of calprotectin. In conclusion, STB could alleviate a decrease of the performance, immune response, and inflammatory response induced by the STEC challenge.

## 1. Introduction

The gastrointestinal (GI) tract digests and absorbs nutrients while also acting as a barrier against harmful substances and pathogens ingested through the diet [1]. Pigs are exposed to a variety of pathogenic challenges, which causes the GI immune system to become activated [2]. Although a highly activated immune system may appear to be the best protective mechanism for animals, it can have a negative impact on animal performance [3]. For example, the overactivation of proinflammatory cytokines such as tumor necrosis factor alpha (TNF-α) and interleukin-6 (IL-6) leads to the poor growth of pigs [4]. Weaned pigs face problems of post-weaning diarrhea (PWD) caused by pathogenic bacteria such as *Shiga toxigenic Escherichia coli* (STEC) and *enterotoxigenic Escherichia coli* (ETEC) during the 4 weeks of the postweaning period [5,6]. The small intestines of pigs show significant structural and functional changes after weaning. These changes lead to the vicissitude of protein conversion rates, microbiota composition, digestive barrier, and immune function [7]. In previous studies, PWD caused a lower growth performance through changes in the gut integrity, microbiome, and villus height, as well as proinflammatory cytokines [8,9]. Many researchers have been studying nonantibiotic biological methods, such as using essential oils, enzymes, probiotics, prebiotics, and organic acids, to prevent digestive diseases by controlling intestinal microbiota [10,11].

Diets in monogastric animals consist mostly of corn and non-starch polysaccharides such as arabinoxylans in Asia [12]. Endo-β-1,4-xylanase (XYL) is a carbohydrate-active enzyme that can hydrolyze the bonds of xylans, thus improving the availability of the antinutritive factors [13]. Xylo-oligosaccharide (XOS) produced through XYL can act as a prebiotic to increase the fermentation metabolites [14]. The concept of stimbiotic (STB), a complex of XYL and XOS, has been recently introduced as a nondigestible and fermentable additive that can improve the fermentation of the fiber microbiome [15,16]. The mechanism of action of STB is that it can stimulate the intestinal microbiota responsible for fiber degradation [17]. In a previous study, Bedford et al. [18] reported that STB can enhance the growth performance and production of short chain fatty acids (SCFAs). Likewise, the supplementation of STB can stimulate the fermentation of dietary fiber, thereby decreasing the digesta viscosity and improving energy utilization [19]. Petry et al. [20] reported that the supplementation of XYL and XOS can improve the intestinal barrier integrity and reduce oxidative stress, respectively. Additionally, poor sanitary conditions, which induce bacterial infection found in industrial pig production, can negatively affect the growth performance and TNF- α, which could alleviate the use of STB [16].

STEC is known to be a major pathogen causing diarrhea, but there is insufficient research validating what causes PWD [21]. Therefore, we conducted experiments to verify the effects of STEC, which induce PWD. In this experiment, we hypothesized that (1) the experimental induction of STEC infection could increase the inflammatory responses and reduce the growth performance of pigs and that (2) the supplementation of STB could attenuate the extent of the performance loss and inflammatory response caused by PWD induced by STEC challenge. Therefore, the purpose of this study was to determine the effects of adding STB on the growth performance, immune response, and inflammatory response when weaned pigs were orally administered with pathogenic *E. coli*.

## 2. Materials and Methods

### 2.1. Ethics

All experimental procedures received prior approval from the Animal Ethics Committee of Chungbuk National University (CBNUA-1618-21-02).

### 2.2. Bacterial Strains, Culture and Challenge

STEC F18 was provided in stock form. The F18 *E. coli* expressed heat labile toxin (LT) and *shiga toxin* type 2e (stx2e). Ten microliter of thawed *E. coli* stock was inoculated into 10 mL of nutrient broth and cultured at 37 °C for 24 h and then subcultured [22]. Thereafter, the subcultured *E. coli* was smeared on MacConkey agar to confirm the bacterial enumeration. A final concentration of 1.2 × 10^10^ CFU/mL was used in this study.

### 2.3. Animals, Experimental Design and Diets

**Figure 1:**
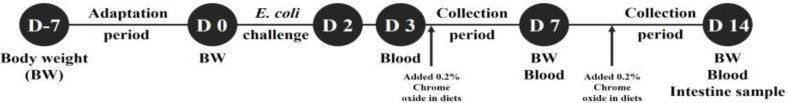
Schematic diagram of the experimental design.

A total of 36 male pigs (Duroc × Yorkshire × Landrace), weaned at 28 d (initial body weight of 8.49 ± 0.10 kg), were assigned to 6 treatments with 6 replicates per treatment. Pigs were individually placed in 45 × 55 × 45 stainless steel metabolism cages in an environmentally controlled room. Pigs were housed in individual pens for 21 days, including 7 days before and 14 days after the first *E. coli* challenge (0 dpi). The experiment was conducted in a 2 × 3 factorial arrangement of treatments consisting of two levels of challenge (challenge and non-challenge) and three levels of STB (0, 0.5, and 1 g/kg diet). Corn and soybean meal basal diets were formulated to meet or exceed the nutrient requirements for the weaned piglets by NRC (Table 1) [23]. The pigs were fed daily at 8:30 and 17:00 h and had ad libitum access to water. Feed residues were removed before the next meal and considered in the calculations. Figure 1 depicts the schematic diagram of the weaned piglets experimental design used for this study. 

### 2.4. Growth Performance

All piglets were weighed every week during the experiment period, and feed consumption was recorded to calculate the average daily gain (ADG), average daily feed intake (ADFI), and gain-to-feed ratio (G:F).

### 2.5. Fecal Scores

The fecal scores were individually recorded at 08:00 and 17:00 by the same person during the entire experimental period. The fecal score was scored using a method used by Zhao et al. [24]. The fecal scores were as follows: 0, Normal feces; 1, Soft feces; 2, Mild diarrhea; and 3, Severe diarrhea.

### 2.6. Nutrient Digestibility

To estimate the digestibility, 0.2% chromium oxide (Cr_2_O_3_) was supplemented with the diets as an indigestible marker. Pigs were fed diets mixed with chromium oxide for 4 consecutive days from 4 dpi to 11, fresh excreta samples were collected in that period. At the end of the experiment, the fecal samples were stored at −20 °C and dried at 70  °C for 72 h and then ground up to pass through a 1-mm screen. All analysis items (feed and fecal) were analyzed for DM and CP. The procedures utilized for the determination of dry matter (DM) and crude protein (CP) digestibility were conducted with the methods by the AOAC [25]. Chromium was analyzed with an ultraviolet absorption spectrophotometer (UV-1201, Shimadzu, Kyoto, Japan). The digestibility was calculated using the following formula: digestibility (%) = [1 − (Nf × Cd)/(Nd × Cf)] × 100, where Nf is the nutrient concentration in feces (% DM), Nd is the nutrient concentration in diet (% DM), Cd is the chromium concentration in diet (% DM), and Cf is the chromium concentration in feces (% DM).

### 2.7. Blood Profile

Blood samples were obtained from the anterior vena cava of 6 pigs per each treatment at 3, 7, and 14 dpi. At the time of collection, blood samples were collected into vacuum tubes containing K3EDTA for CBC analysis and nonheparinized tubes for serum analysis, respectively. After collection, blood samples were centrifuged (3000× *g* for 15 min at 4 °C). The white blood cells (WBC), basophils, neutrophils, and lymphocyte levels in the whole blood were measured using an automatic blood analyzer (ADVIA 120, Bayer, NY, USA). The immunoglobulin G (IgG) and immunoglobulin A (IgA) levels were gauged using an automatic biochemistry blood analyzer (Hitachi 747; Hitachi, Tokyo, Japan).

### 2.8. Morphological Analysis of Small Intestine

At the end of the experiment (14 dpi), the pigs were anesthetized with carbon dioxide gas after blood sampling and euthanized by exsanguination. Intestinal tissues of about 10 cm from the ileum (close to the ileocecal junction), were collected and fixed in 10% neutral buffered formalin (NBF; Sigma-Aldrich, St. Louis, MO, USA). After cutting the intestine sample, it was dehydrated and dealcoholized. The samples were then installed on slides, treated with paraffin, and stained with hematoxylin and eosin (ab245880, abcam). Villus height and crypt depth were measured under a light microscope (OLYMPUS DP71, BX50F-3, Olympus Optical Co. Ltd., Tokyo, Japan). Villus height (VH) was determined by measuring the distance between the tip of the villi to the villus crypt junction, and the crypt depth (CD) was determined by measuring the distance between adjacent villi. Mean values of 10 fields, 30 well-oriented, complete villus-crypt structures were calculated for each pig.

### 2.9. Measurements of Pro-Inflammatory Cytokine

The inflammatory biomarkers such as interleukin-6 (IL-6) and tumor necrosis factor α (TNF-α) were measured using commercially available ELISA kits according to manufacturer’s instructions (Quantikine, R&D Systems, Minneapolis, MN, USA).

### 2.10. Expression of Tight Junction Proteins

The expression of claudin-1 and calprotectin was determined via immunohistochemistry. Histologic tissues were deparaffinized, rehydrated, and rinsed using standard methods. The sections of slide were incubated with the primary antibody for claudin-1 (1:200; Novus Biologicals, Minneapolis, MN, USA) and calprotectin (1:800; Thermo Fisher Scientific, Waltham, MA, USA), followed by washing and incubation with the secondary antibody envision anti-rabbit for claudin-1 (Dako, Santa Clara, CA, USA) and calprotectin (Dako, Santa Clara, CA, USA) for 30 min. The stained samples were evaluated under a microscope (Axio Scan Z1; Carl Zeiss, Jena, Germany), and the images were analyzed via Zen 3.4 blue edition.2.11. Statistical Analysis.

Data for the effects of different levels of STB were added with a challenge or not. Data were subjected to two-way ANOVA. All data were statistically analyzed with a PROC General Linear Models (GLM) of SAS (SAS Institute, Cary, NC, USA). Differences between treatment groups were measured using Duncan’s multiple range test, with a *p*-value of less than 0.05 designating statistical significance.

## 3. Results

### 3.1. Growth Performance

A difference was observed in the BW on 7 dpi and 14 dpi among the treatments (Table 2). The challenged groups showed lesser (*p* < 0.01) BW compared with the non-challenged groups, and the supplementation of STB groups showed greater (*p* < 0.05) BW. STB supplementation showed greater (*p* < 0.05) on ADG in the whole experiment period. Additionally, the supplementation of STB showed greater (*p* < 0.05) G:F on 0–7 d and 0–14 d. There were interactions among the treatments and challenges in the BW, ADFI, and ADG. Supplementations STB 0.5 g/kg (STB5) and STB 1 g/kg (STB10) improved the BW (*p* < 0.01), ADFI (*p* < 0.01), and ADG (*p* < 0.01) in piglets challenged with *E. coli*.

### 3.2. Fecal Score

The fecal score was greater (*p* < 0.05) in the challenged treatments compared with the non-challenged treatments on 1–7 dpi and 1–14 dpi (Table 3). There was no interaction among the treatments and challenge.

### 3.3. Nutrient Digestibility

The digestibility of DM showed a significant increase (*p* < 0.05) in the supplementations of STB5 and STB10 compared with the non-supplementation of STB (Table 4). The digestibility of DM and CP were significantly decreased (*p* < 0.05) and challenged with STEC on 14 dpi. There was no interaction among the treatments and challenge.

### 3.4. Blood Profile

Table 5 showed the results of blood profiles at 3, 7, and 14 dpi. At 3 dpi, the counts of the white blood cells were lesser (*p* < 0.05) in the challenged groups compared to 3 dpi and 7 dpi (Table 5). Neutrophils showed up more in the challenged groups compared to the non-challenged groups on 3, 7, and 14 dpi. Lymphocytes showed up less (*p* < 0.05) in the challenged groups compared to the non-challenge groups on 3, 7, and 14 dpi. The supplementations of STB5 and STB10 showed reduced (*p* < 0.05) WBC, neutrophils, and greater (*p* < 0.05) lymphocytes compared to the non-supplementation of the STB group. There were interactions among the treatments and challenges in neutrophils and lymphocytes. Supplementations STB5 and STB10 improved (*p* < 0.01) the lymphocytes and neutrophils in piglets challenged with *E. coli* on d 14.

### 3.5. Measurements of Proinflammatory Cytokine and Immunoglobulin

The non-challenged treatments were greater (*p* < 0.05) than the challenged treatments in IL-6 on 3 dpi (Table 6). The supplementations of STB5 and STB10 decreased (*p* < 0.05) the TNF-α on 3 dpi. On 7 and 14 dpi compared to non-supplementation of the STB group. There were interactions among the treatments and challenges in TNF-α. IgG showed more in the challenged groups compared with the non-challenged groups on 3, 7, and 14 dpi. Supplementations STB5 and STB10 improved (*p* < 0.01) TNF-α in piglets challenged with *E. coli*.

### 3.6. Morphological Analysis of Small Intestine

**Figure 2:**
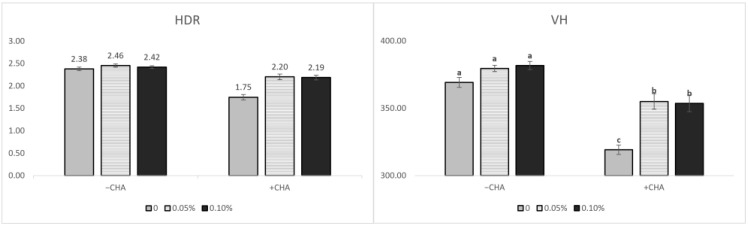
Effects of stimbiotic on the villus height (VH) and height-to-depth ratio (HDR) in the ileum of weaned piglets. Data are represented as the mean ± standard error. ^a,b,c^ Different superscript letters indicate significant differences between groups (*p* < 0.05).

**Figure 3:**
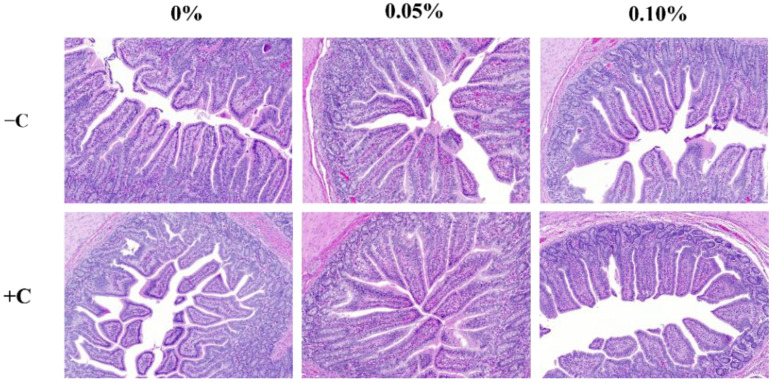
Histological analysis of the intestinal morphology of weaned piglets. Figures display the morphology of ileum tissue from pigs in six dietary treatments.

The non-challenged treatments were longer (*p* < 0.05) than the challenged treatments in VH. CD was greater (*p* < 0.05) in the challenged treatments than the non-challenged treatments (Table 7). HDR was greater (*p* < 0.05) in the non-challenged treatments than the challenged treatments. The supplementation of STB5 and STB10 was increased the VH and HDR (*p* < 0.05) compared with the non-supplementation of STB group (Figure 2). There was an interaction among the treatments and challenges in the VH, CD, and HDR. Supplementations STB5 and STB10 improved the VH (*p* < 0.01) and HDR (*p* < 0.01) in piglets challenged with *E. coli*. Representative images of the intestinal morphology are captured in Figure 3.

### 3.7. Expression of Tight Junction Proteins

**Figure 4:**
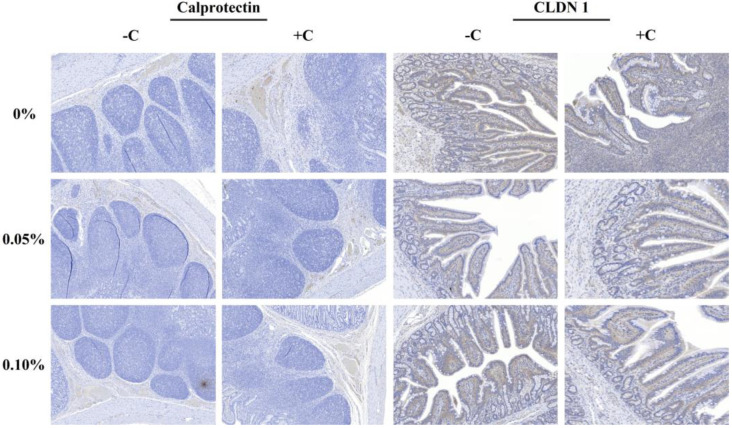
Immunohistochemistry staining of the protein expression for Claudin-1 and Calprotectin of the ileum tissue. Positive staining indicated by a brown precipitate in cells.

The non-challenged treatments were greater than the challenged treatments in the stained area of calprotectin (Calp) and claudin-1 (CLDN-1) (*p* < 0.05), and the supplementation with STB5 tended to reduce (*p* = 0.052) the stained area of the Calp (Table 8). However, the supplementation of STB did not affect CLDN-1. There was no interaction among the treatments and challenge. Representative images of immunohistochemistry staining of the protein expression are captured in Figure 4.

## 4. Discussion

The small intestine is a major area to digest and absorb nutrients. It can serve as the first line of defense against various harmful substances or pathogens [26]. Weaning causes numerous changes, including enzymatic, morphological, and inflammatory changes that can, damage the intestinal integrity [27]. Harmful bacterial pathogens (i.e., *Escherichia coli*) can invade through the damaged intestine and lead to decreased nutrient digestion and absorption, consequently decreasing the growth rate [28,29]. In the present study, the STEC challenge decreased the growth performance. The ADG and G:F were reduced by 31% and 23%, respectively. These results were agreement with the results of He et al. [30], which reported that the challenge with *E. coli* decreased the BW, ADG, ADFI, and G:F compared to the non-challenged groups. A previous study showed that *E. coli* infection can increase the frequency of diarrhea [31,32]. This result is similar to those obtained in prior studies using an *E. coli* strain to inoculate pigs. In the present study, the VH and HDR were decreased, but the CD was increased in STEC-challenged pigs. These results are consistent with previous studies showing that pigs challenged with *E. coli* had a lower VH and HDR in the small intestine [29,33,34,35]. The *E. coli* challenge could cause a defect of the intestinal barrier integrity by downregulating the expression of tight junction (TJ) proteins. [36,37]. The STEC challenge significantly decreased the CLDN-1 and Calp expression compared to the non-challenge groups in the present, as in the previous study of Yu et al. [34]. TJ proteins, located in the intercellular structure, are junctional adhesion molecules and multi-protein complexes composed of transmembrane proteins [38,39]. They are commonly considered as a strong barrier against the absorption of endotoxin [39]. These results indicated that the oral administration of STEC successfully induced the PWD model, as we hypothesized before starting this study.

STB is a fermentable additive that can stimulate the development of a proportion of bacterial species involved in fiber degradation [16,20]. For example, XOS can improve the intestinal morphology and expression of TJ proteins by improving the gut microbiota communities [40]. Thus, we hypothesized that dietary STB supplementation could mitigate pathogenic *E. coli*-induced intestinal damage by improving the barrier integrity and suppressing inflammation in weaned pigs. In our study, the supplementation of STB improved the intestinal morphology and expression of TJ in pigs challenged with STEC. These results were in agreement with previous results showing that 0.01% and 0.05% XOS could increase the VH in the ileum and HDR in the jejunum [40,41]. Consistently, prebiotics (i.e., alginate-oligosaccharide, AOS; mannan-oligosaccharide, MOS) could mitigate the intestinal mucosa injury by improving the VH and HDR [42,43]. Additionally, the supplementation of MOS could elevate the expression levels of TJ proteins such as zonula occludens-1, CLDN-1, and Occludin reduced by the ETEC challenge [43]. These results were similar to the results of the present study. Previous studies have shown that prebiotics could upregulate the expression levels of the intestinal TJ proteins in piglets [44,45]. Through many studies, including ours, the consistent improvement of oligosaccharides might be associated with the production of SCFAs. SCFAs are volatile fatty acids (VFA). They mainly include acetic acid, propionic acid, and butyric acid [46]. SCFAs can promote the intestinal morphology and TJ proteins of broilers [47,48]. They can also be used as energy sources, leading to an improved absorption surface in the intestine via increased the proliferation of epithelial cells [49,50,51,52]. The exact mechanism of action of STB is currently unknown. It has been suggested that the improvement of the gut integrity is due to improved gut fermentation.

In the current study, STB supplementation alleviated the reduction of nutrient digestibility and growth performance caused by the STEC challenge. This effect of STB might be due to its ability to improve gut health, as mentioned above. It might also be due to its ability to improve the enzyme activities [53]. Previous studies have reported that MOS supplementation can increase the mucosal enzyme activities, including duodenal sucrase, ileal lactase, and ileal maltase activities in the ETEC challenged pigs [43].

The count of WBC is one of the most common diagnostic indications of infection. WBC are an important part of the immune system that fights against infections in the body [54]. While neutrophils are the first cells to move into infected tissues during inflammatory reactions and phagocytose bacteria with their particles, lymphocytes produce specialized cellular and humoral immune responses [55]. The neutrophils-to-lymphocytes ratio is often used as a biomarker to assess the systemic inflammation severity. According to Liu et al. [31], *E. coli* can cause inflammation in weaned piglets by boosting WBC counts and neutrophils. A previous study showed that Chito-oligosaccharides with a function similar to XOS can mitigated the increase in the value of neutrophils [33]. In the current study, during *E. coli* infection, pigs supplemented with STB had a lower neutrophils-to-lymphocytes ratio than non-supplemented with the STB groups, indicating that inflammation was decreased in piglets with STB supplementation. These results indicate that STB can reduce *E. coli*-induced intestinal inflammation in pigs potentially by lowering the bacterial growth and metabolism in gut bacterial environments [56].

When *E. coli* enters the bloodstream, the general immune response is triggered, and immune cells in tissues are activated by bacterial ligands, resulting in a fast burst of proinflammatory cytokines [57]. In a previous study, acute exposure to *E. coli* can increase the blood endotoxin levels, leukocyte numbers, and proinflammatory cytokine production [58]. *Shiga toxins* released by pathogenic *E. coli* could cause systemic inflammation, therefore increasing inflammatory cytokines [59]. Moreover, STEC could utilize hemoglobin as an iron source for their proliferation and virulence production [60]. A recent study showed that *E. coli* can increase the production of proinflammatory cytokines in piglets [61]. Yu et al. [43] reported that the supplementation of MOS can reduce the concentrations of inflammatory cytokines. Our study also showed that supplementation with STB reduced the levels of the proinflammatory cytokines. These results confirmed that the *E. coli* challenge induces elevated concentrations of inflammatory cytokines and that the supplementation with STB decreases the concentrations of inflammatory cytokines in piglets.

## 5. Conclusions

The challenge with *E. coli* decreased the growth performance and VH, increased the inflammatory response, and downregulated TJ proteins. However, supplementing STB, a complex of XYL and XOS, alleviated these negative effects of the *E. coli* challenge in this study. Supplementation with STB improved the growth performance, intestinal morphology, and inflammatory responses. These results suggest that STB might be effective in mitigating post-weaning diarrhea, a severe disease in weaning piglets.

## Figures and Tables

**Table 1:** Compositions of basal diets (as-fed basis).

Items	Content
Ingredients, %	
Corn	34.43
Extruded corn	15.00
Lactose	10.00
Dehulled soybean meal, 51% CP ^1^	13.50
Soy protein concentrate, 65% CP ^1^	10.00
Plasma powder	6.00
Whey	5.00
Soy oil	2.20
Monocalcium phosphate	1.26
Limestone	1.40
L-Lysine-HCl, 78%	0.06
DL-Methionine, 50%	0.15
Choline chloride, 25%	0.10
Vitamin premix ^2^	0.25
Trace mineral premix ^3^	0.25
Salt	0.40
Total	100.00
Calculated value	
ME, Kcal/kg	3433
CP, %	20.76
Lysine, %	1.35
Methionine, %	0.39
Ca	0.82
P	0.65
Analyzed value	
ME, kcal/kg	3512
CP, %	20.92

^1^ CP, crude protein. ^2^ Provided per kg of complete diet: vitamin A, 11,025 IU; vitamin D_3_, 1103 IU; vitamin E, 44 IU; vitamin K, 4.4 mg; riboflavin, 8.3 mg; niacin, 50 mg; thiamine, 4 mg; d-pantothenic, 29 mg; choline, 166 mg; and vitamin B12, 33 mg. ^3^ Provided per kg of complete diet without zinc: Cu (as CuSO_4_*5H_2_O), 12 mg; Mn (as MnO_2_), 8 mg; I (as KI), 0.28 mg; and Se (as Na_2_SeO_3_*5H_2_O), 0.15 mg.

**Table 2:** Effects of stimbiotic supplementation on the growth performance in pigs challenged with STEC.

Items, kg	−C			+C			SE ^1^	C		STB			*p*-Value		
	0	0.05	0.1	0	0.05	0.1		−	+	0	0.05	0.1	C	STB	C × STB
BW															
d − 7	8.46	8.46	8.46	8.47	8.47	8.46	0.08	8.46	8.47	8.47	8.47	8.46	0.95	0.99	1.00
d 0	9.92	10.33	10.32	9.97	10.30	10.28	0.18	10.19	10.18	9.94	10.32	10.30	0.96	0.08	0.96
d 7	11.85	12.53	12.35	10.52	11.52	11.68	0.22	12.24	11.24	11.18 ^b^	12.03 ^a^	12.02 ^a^	<0.01	<0.01	0.32
d 14	14.55 ^b^	15.50 ^a^	15.25 ^a^	12.37 ^c^	14.15 ^b^	14.10 ^b^	0.20	15.10	13.54	13.46 ^b^	14.83 ^a^	14.68 ^a^	<0.01	<0.01	0.04
Pre															
ADG	0.21	0.27	0.27	0.21	0.26	0.26	0.02	0.25	0.25	0.21 ^b^	0.26 ^a^	0.26 ^a^	0.93	0.03	0.95
ADFI	0.32	0.36	0.37	0.32	0.38	0.36	0.02	0.35	0.35	0.32 ^b^	0.37 ^a^	0.37 ^a^	0.96	0.02	0.76
G:F	0.66	0.73	0.70	0.69	0.68	0.73	0.04	0.69	0.70	0.67	0.71	0.71	0.90	0.51	0.53
0–1 W															
ADG	0.28	0.31	0.29	0.08	0.17	0.20	0.03	0.29	0.15	0.18 ^b^	0.24 ^a^	0.25 ^a^	<0.01	0.02	0.13
ADFI	0.42	0.45	0.44	0.32	0.36	0.38	0.02	0.44	0.35	0.37	0.41	0.41	<0.01	0.08	0.49
G:F	0.66	0.69	0.67	0.25	0.50	0.52	0.06	0.67	0.42	0.46 ^b^	0.59 ^a^	0.59 ^a^	<0.01	0.04	0.07
1–2 W															
ADG	0.39	0.42	0.41	0.26	0.38	0.35	0.03	0.41	0.33	0.33 ^b^	0.40 ^a^	0.38 ^ab^	<0.01	0.04	0.44
ADFI	0.61 ^ab^	0.59 ^ab^	0.58 ^ab^	0.51 ^c^	0.62 ^a^	0.57 ^b^	0.01	0.59	0.57	0.56 ^b^	0.61 ^a^	0.58 ^ab^	0.03	0.01	<0.01
G:F	0.63	0.72	0.71	0.52	0.61	0.60	0.04	0.69	0.58	0.57	0.66	0.66	0.01	0.10	1.00
0–2 W															
ADG	0.33 ^a^	0.37 ^a^	0.35 ^a^	0.17 ^c^	0.28 ^b^	0.27 ^b^	0.01	0.35	0.24	0.25 ^b^	0.32 ^a^	0.31 ^a^	<0.001	<0.01	0.02
ADFI	0.49 ^ab^	0.52 ^a^	0.50 ^a^	0.38 ^c^	0.46 ^b^	0.47 ^b^	0.01	0.50	0.44	0.44 ^b^	0.49 ^a^	0.48 ^a^	<0.001	<0.01	<0.01
G:F	0.68	0.72	0.71	0.45	0.60	0.59	0.03	0.70	0.54	0.56 ^b^	0.66 ^a^	0.65 ^a^	<0.001	0.01	0.20

Abbreviations: -C: non-challenge with STEC; +C: challenge with STEC; 0, 0.5, and 0.1: supplementation of STB 0, 0.05%, and 0.1%, respectively; BW: body weight; ADG: average daily gain; ADFI: average daily feed intake; G:F: gain-to-feed ratio; Pre: pre-inoculation; and ^l^ SE, standard error. ^a,b,c^ Values within a row with different superscripts are significantly different.

**Table 3:** Effects of stimbiotic on the fecal score ^1^ of pigs challenged with STEC.

Items	−C			+C			SE ^2^	C		STB			*p*-Value		
	0	0.05	0.10	0	0.05	0.10		−	+	0	0.05	0.10	C	STB	C × STB
Day −6 to 0	1.90	1.69	1.57	1.69	1.60	1.74	0.18	1.72	1.68	1.80	1.64	1.66	0.76	0.65	0.56
Day 1 to 7	0.86	0.46	0.61	1.11	0.98	1.05	0.18	0.64	1.05	0.98	0.72	0.83	0.01	0.35	0.74
Day 8 to 14	0.25	0.11	0.23	0.36	0.33	0.27	0.08	0.20	0.32	0.30	0.22	0.25	0.07	0.56	0.50
Day 1 to 14	0.54	0.29	0.42	0.72	0.67	0.68	0.12	0.42	0.69	0.63	0.48	0.55	0.01	0.42	0.70

Abbreviations: −C: non-challenge with STEC; +C: challenge with STEC; 0, 0.5, and 0.1: supplementation of STB 0, 0.05%, and 0.1%, respectively ^1^ fecal score, 0 = firmed faces; 1 = slightly soft faces; 2 = soft formed faces; 3 = diarrhea; and ^2^ SE, standard error.

**Table 4:** Effects of stimbiotic supplementation on the nutrient digestibility of pigs challenged with STEC.

Items	−C			+C			SE ^1^	C		STB			*p*-Value		
	0	0.05	0.1	0	0.05	0.1		−	+	0	0.05	0.1	C	STB	C × STB
1 W															
DM	83.17	84.08	83.85	81.42	83.43	82.90	0.25	83.70	82.58	82.29 ^b^	83.76 ^a^	83.38 ^a^	<0.001	<0.001	0.10
CP	74.55	75.45	74.82	74.45	75.22	74.67	0.70	74.94	74.78	74.50	75.33	74.74	0.78	0.48	1.00
2 W															
DM	83.52	84.10	83.70	82.50	83.10	82.83	0.26	83.77	82.81	83.01 ^b^	83.60 ^a^	83.27 ^ab^	<0.001	0.09	0.95
CP	75.78	76.35	76.07	74.78	75.20	75.05	0.43	76.07	75.01	75.28	75.78	75.59	0.01	0.51	0.98

Abbreviations: −C: non-challenge with STEC; +C: challenge with STEC; 0, 0.5, and 0.1: supplementation of STB 0, 0.05%, and 0.1%, respectively; DM: dry matter; CP: crude protein; and ^1^ SE, standard error. ^a,b^ Values within a row with different superscripts are significantly different.

**Table 5:** Effects of stimbiotic supplementation on the blood profile of pigs challenged with STEC.

Items	−C			+C			SE ^1^	C		STB			*p*-Value		
	0	0.05	0.1	0	0.05	0.1		−	+	0	0.05	0.1	C	STB	C × STB
D 3															
WBC, 10³/µL	21.42	17.32	17.87	24.22	22.12	22.90	0.67	18.87	23.08	22.82 ^a^	19.72 ^b^	20.38 ^b^	<0.01	<0.01	0.20
Bas, %	0.43	0.40	0.50	0.45	0.48	0.55	0.06	0.44	0.49	0.44	0.44	0.53	0.30	0.27	0.85
Neu, %	52.28	50.48	50.60	57.98	54.33	54.35	0.63	51.12	55.56	55.13 ^a^	52.41 ^b^	52.48 ^b^	<0.01	<0.01	0.23
Lym, %	40.20 ^a^	41.43 ^a^	40.82 ^a^	33.62 ^c^	37.67 ^b^	37.57 ^b^	0.63	40.82	36.28	36.91 ^b^	39.55 ^a^	39.19 ^a^	<0.01	<0.01	0.03
D7															
WBC, 10³/µL	18.67	15.82	16.35	21.55	19.07	19.22	0.37	16.94	19.94	20.11 ^a^	17.44 ^b^	17.78 ^b^	<0.01	<0.01	0.85
Bas, %	0.80	0.60	0.60	0.67	0.70	0.67	0.06	0.67	0.68	0.73	0.65	0.63	0.82	0.20	0.11
Neu, %	50.83	49.73	49.55	53.60	52.25	51.82	0.37	50.04	52.56	52.22 ^a^	50.99 ^b^	50.68 ^b^	<0.01	<0.01	0.80
Lym, %	40.75	41.77	42.02	38.28	40.15	40.45	0.50	41.51	39.63	39.52 ^b^	40.96 ^a^	41.23 ^a^	<0.01	<0.01	0.61
D14															
WBC, 10³/µL	16.30	16.08	16.30	16.73	16.38	16.50	0.35	16.23	16.54	16.52	16.23	16.40	0.28	0.71	0.94
Bas, %	0.60	0.55	0.70	0.67	0.50	0.57	0.09	0.62	0.58	0.63	0.53	0.63	0.60	0.40	0.55
Neu, %	40.20 ^c^	40.23 ^c^	40.72 ^c^	47.07 ^a^	42.37 ^b^	42.68 ^b^	0.40	40.38	44.04	43.63 ^a^	41.30 ^b^	41.70 ^b^	<0.01	<0.01	<0.01
Lym, %	51.93 ^a^	51.42 ^a^	51.77 ^a^	45.13 ^c^	49.00 ^b^	49.38 ^b^	0.43	51.71	47.84	48.53 ^b^	50.21 ^a^	50.58 ^a^	<0.01	<0.01	<0.01

Abbreviations: −C: non-challenge with STEC; +C: challenge with STEC; 0, 0.5, and 0.1: supplementation of STB 0, 0.05%, and 0.1%, respectively; WBC: White blood cells; Bas; Basophils; Neu: Neutrophils; and Lym; Lymphocytes. ^1^ SE, standard error. ^a,b,c^ Values within a row with different superscripts are significantly different.

**Table 6:** Effects of stimbiotic supplementation on the proinflammatory cytokines of pigs challenged with STEC.

Items	−C			+C			SE^1^	C		STB			*p*-Value		
	0	0.05	0.1	0	0.05	0.1		−	+	0	0.05	0.1	C	STB	C × STB
D3															
TNFα	32.8 ^b^	31.7 ^b^	34.7 ^b^	47.7 ^a^	31.3 ^b^	32.9 ^b^	2.95	33.0	37.3	40.25 ^a^	31.48 ^b^	33.8 ^b^	0.09	0.02	0.01
IL-6	169.1	162.2	164.5	661.5	626.1	636.1	28.14	165.3	641.2	415.3	394.2	400.3	<0.01	0.74	0.87
IgG	205.3	183.8	194.5	241.0	210.5	214.3	15.00	194.6	221.9	223.2 ^a^	197.2 ^b^	204.4 ^b^	<0.01	0.01	0.63
IgA	1.0	1.5	1.5	1.3	1.3	1.0	0.21	1.3	1.2	1.2	1.4	1.3	0.51	0.48	0.14
D7															
TNFα	29.2	26.3	27.2	31.9	28.9	28.4	0.65	27.6	29.7	30.6 ^a^	27.6 ^b^	27.8 ^b^	<0.01	<0.01	0.45
IL-6	106.3	92.8	95.9	356.1	312.7	315.0	8.21	98.3	327.9	231.2 ^a^	202.8 ^b^	205.4 ^b^	<0.01	<0.01	0.12
IgG	166.2	142.7	156.0	179.2	174.2	173.5	4.11	154.9	175.6	172.7 ^a^	158.4 ^b^	164.8 ^ab^	<0.01	0.01	0.08
IgA	1.0	1.0	1.5	1.0	1.2	1.2	0.13	1.2	1.1	1.00 ^b^	1.1 ^ab^	1.3 ^a^	0.61	0.05	0.18
D14															
TNFα	26.7	21.0	23.1	29.3	25.5	27.6	0.77	23.6	27.5	28.0 ^a^	23.2 ^c^	25.4 ^b^	<0.01	<0.01	0.33
IL-6	57.5	43.8	45.8	86.7	72.9	79.0	1.95	49.0	79.5	72.1 ^a^	58.3 ^c^	62.4 ^b^	<0.01	<0.01	0.50
IgG	146.2	143.8	146.0	174.8	161.8	163.0	5.81	145.3	166.6	160.5	152.8	154.5	<0.01	0.39	0.55
IgA	1.0	1.0	1.0	1.2	1.0	1.2	0.10	1.0	1.1	1.1	1.0	1.1	0.17	0.61	0.61

Abbreviations: −C: non-challenge with STEC; +C: challenge with STEC; 0, 0.5, and 0.1: supplementation of STB 0, 0.05%, and 0.1%, respectively; TNFα: tumor necrosis factor alpha; IL-6: interleukin-6, IgG: Immunoglobulin G; and IgA: Immunoglobulin A. ^1^ SE, standard error. ^a,b,c^ Values within a row with different superscripts are significantly different.

**Table 7:** Effects of stimbiotic supplementations on the villus height and crypt depth of pigs challenged with STEC.

Items	−C			+C			SE ^1^	C		STB			*p*-value		
	0	0.05	0.1	0	0.05	0.1		−	+	0	0.05	0.1	C	STB	C × STB
VH	369.22 ^a^	379.63 ^a^	381.80 ^a^	319.15 ^c^	355.06 ^b^	353.71 ^b^	4.33	376.88	342.64	344.19 ^b^	367.35 ^a^	367.75 ^a^	<0.01	<0.01	0.013
CD	155.30 ^b^	154.72 ^b^	157.91 ^b^	183.60 ^a^	161.37 ^b^	161.78 ^b^	3.29	155.98	168.92	169.45 ^a^	158.05 ^b^	159.84 ^b^	<0.01	<0.01	<0.01
HDR	2.38 ^a^	2.46 ^a^	2.42 ^a^	1.75 ^c^	2.20 ^b^	2.19 ^b^	0.05	2.42	2.05	2.07 ^b^	2.33 ^a^	2.3 ^a^	<0.01	<0.01	<0.01

Abbreviations: −C: non-challenge with STEC; +C: challenge with STEC; 0, 0.5, and 0.1: supplementation of STB 0, 0.05%, and 0.1%, respectively; VH: villus height; CD: crypt depth; and HDR: height-to-depth ratio. ^1^ SE, standard error. ^a,b,c^ Values within a row with different superscripts are significantly different.

**Table 8:** Effects of stimbiotic supplementation on the expression of tight junction proteins of pigs challenged with STEC.

Items	−C			+C			SE ^1^	C		STB			*p*-Value		
	0	0.05	0.10	0	0.05	0.10		−	+	0	0.05	0.10	C	STB	C × STB
Calp	0.03	0.02	0.04	0.09	0.06	0.06	0.010	0.03	0.07	0.06	0.04	0.05	<0.01	0.052	0.080
CLDN-1	19.43	17.24	18.27	13	14.83	15.12	1.100	18.31	14.31	16.21	16.03	16.69	<0.01	0.830	0.170

Abbreviations: −C: non-challenge with STEC; +C: challenge with STEC; 0, 0.5, and 0.1: supplementation of STB 0, 0.05%, and 0.1%, respectively; Calp: Calprotectin; and CLDN-1: Claudin-1. ^1^ SE, standard error.

## Data Availability

Data sharing is not applicable to this article.
